# Effects of Post-Exercise Whey Protein Consumption on Recovery Indices in Adolescent Swimmers

**DOI:** 10.3390/ijerph17217761

**Published:** 2020-10-23

**Authors:** Brandon J. McKinlay, Alexandros Theocharidis, Tony Adebero, Nigel Kurgan, Val A. Fajardo, Brian D. Roy, Andrea R. Josse, Heather M. Logan-Sprenger, Bareket Falk, Panagiota Klentrou

**Affiliations:** 1 Department of Kinesiology, Faculty of Applied Health Sciences, Brock University, St. Catharines, ON L2S 3A1, Canada; brandon.mckinlay@brocku.ca (B.J.M.); theocharidisalex@gmail.com (A.T.); tadebero@uwo.ca (T.A.); nk10gw@brocku.ca (N.K.); vfajardo@brocku.ca (V.A.F.); broy@brocku.ca (B.D.R.); bfalk@brocku.ca (B.F.); 2 Centre for Bone and Muscle Health, Faculty of Applied Health Sciences, Brock University, St. Catharines, ON L2S 3A1, Canada; ajosse@yorku.ca; 3 School of Kinesiology and Health Science, Faculty of Health, York University, Toronto, ON M3J 1P3, Canada; 4 Canadian Sport Institute Ontario, 857 Morningside Avenue, Toronto, ON M1C 0C7, Canada; Heather.Sprenger@uoit.ca; 5 Faculty of Health Sciences, Ontario Tech University, Oshawa, ON L1G 0C5, Canada

**Keywords:** inflammation, cytokines, muscle damage, muscle soreness, swimming performance, youth athletes, high-intensity interval swimming

## Abstract

*Purpose*: This study examined the effect of whey protein consumption following high-intensity interval swimming (HIIS) on muscle damage, inflammatory cytokines and performance in adolescent swimmers. *Methods*: Fifty-four swimmers (11–17 years-old) were stratified by age, sex and body mass to a whey protein (PRO), isoenergetic carbohydrate (CHO) or a water/placebo (H_2_O) group. Following baseline blood samples (06:00 h) and a standardised breakfast, participants performed a maximal 200 m swim, followed by HIIS. A total of two post-exercise boluses were consumed following HIIS and ~5 h post-baseline. Blood and 200 m performance measurements were repeated at 5 h, 8 h and 24 h from baseline. Muscle soreness was assessed at 24 h. Creatine kinase (CK), interleukin-6 (IL-6), interleukin-10 (IL-10) and tumor necrosis factor-alpha (TNF-α) were measured in plasma. *Results*: No difference in 200 m swim performance was observed between groups. CK activity was elevated at 5 h compared to baseline and 24 h and at 8 h compared to all other timepoints, with no differences between groups. Muscle soreness was lower in PRO compared to H_2_O (*p* = 0.04). Anti-inflammatory IL-10 increased at 8 h in PRO, while it decreased in CHO and H_2_O. *Conclusions*: Post-exercise consumption of whey protein appears to have no additional benefit on recovery indices following HIIS compared to isoenergetic amounts of carbohydrate in adolescent swimmers. However, it may assist with the acute-inflammatory response.

## 1. Introduction

Youth (i.e., 10–17 years-old) swimming competitions often require athletes to participate in several events over multiple days, performing one or more of the four swim-strokes at varying distances (50–1500 m), across multiple heats, semi-finals and finals [1]. The high number of maximal effort swims, coupled with short recovery times between events and multiple warm-ups and cool-downs may challenge fuel stores [1] and may result in some damage to muscle tissue as reflected by small increases in creatine kinase [2], possibly affecting force generating capacity. As a result, the competitive swim environment can produce performance decrements, which can potentially be magnified the deeper an athlete progresses in competition (e.g., semi-finals and finals) [1,3]. Therefore, determining interventions that can reduce the magnitude of performance decrement could be beneficial for the youth athlete. 

Nutritional interventions such as the consumption of protein following intense exercise is important in the restoration of functional capacity. For example, in adults the consumption of protein following intense exercise has been used to reduce the extent of muscle damage [4,5,6] and as a temporary source of energy [7]. Additionally, post-exercise consumption of protein may improve anti-inflammatory signaling through the downregulation of nuclear factor kappa-β (NF_K_β) signaling pathway, reducing the magnitude of pro-inflammatory cytokines (e.g., interleukin 6, interleukin 1 and tumor necrosis factor’s), potentially expediting the process of tissue repair and regeneration [8,9]. However, previous studies which reported an attenuated pro-inflammatory response post exercise with the consumption of protein did not reassess performance to confirm its meaningfulness with respect to performance recovery [10,11]. Furthermore, previous studies of post-exercise protein consumption and its effects on acute recovery are restricted to adult athletes, with little to no available literature in youth athletes.

Whey protein, a milk protein subfraction, contains all essential amino-acids and is characterized by its quick digestion and absorption [12]. This unique attribute allows for a rapid delivery of amino acids to skeletal muscle to be used in the stimulation of muscle protein synthesis and reduction of protein breakdown, presumably optimizing the tissue repair and regeneration process [13], or used to assist in the resynthesis of glycogen stores when adequate amounts of carbohydrate are not readily available [14]. Based on these characteristics, whey protein may prove to be a viable option to hasten recovery between events, potentially attenuating performance decrements in swimming. Although literature related to the use of whey protein as a recovery aid is currently limited in swimmers, Cade and colleagues [15] observed that the consumption of a milk protein or a carbohydrate supplement before and following an intense pool training session resulted in a quicker return to pre-exercise levels of creatine kinase and lactate dehydrogenase (muscle damage markers) compared to water, possibly reflecting a faster repair. However, it was not clear whether the two supplementation protocols were isoenergetic. Therefore, it is difficult to discern whether the recovery observed was related to energy provision. There are several studies which have examined the acute effects of post-exercise isolated protein consumption on recovery. However, these focus largely on resistance training or on weight-bearing activities, with little focus on non-weight-bearing aerobic activities, such as swimming [16]. Furthermore, the effects of protein consumption following intense exercise in adolescent athletes has not been examined. As adolescent athletes have higher protein requirements due to growth and development demands [17], it cannot be assumed that post-exercise protein consumption will have similar effects in youth athletes as observed in adult athletes. 

The purpose of the present study was to examine the effect of whey protein consumption following a high-intensity interval swim session (HIIS) on subsequent performance, muscle soreness, plasma creatine kinase (CK) levels and inflammatory cytokines, compared with isoenergetic carbohydrate and flavoured water in the acute (0–8 h) and short-term (8–24 h) recovery periods. It is hypothesized that whey protein consumption will result in better performance-related recovery, lower muscle soreness, and lower plasma CK activity and cytokines, compared with both carbohydrates and placebo.

## 2. Materials and Methods

### 2.1. Participants

A total of 60 male and female competitive adolescent (11–17 years-old) swimmers were recruited to participate in this study, designed to examine the effect of post-exercise whey protein consumption on subsequent exercise performance, muscle damage and inflammation. Of this original cohort, 54 male (*n* = 26) and female (*n* = 28) had complete performance, muscle damage and inflammation data and were included in the final analysis. All participants and their parents/guardians received a thorough explanation of the study’s purpose, procedures, benefits, and potential risks, and consent was obtained from both the participant and their parents/guardians prior to study commencement. In addition, all participants completed a medical screening questionnaire to report injuries, allergies and/or health related conditions, as well as a training and sport history questionnaire. This training and sports history questionnaire required participants to describe the level of competition, the number of years participating, training volume per week and perceived intensity of the given sport or training modality. To participate in the study, all swimmers were required to train ≥5 sessions/week and to have competed for a minimum four years. Additionally, participants were required to be free of any musculoskeletal injuries and medical conditions that would have prevented them from participating in maximal exercise and were not currently taking any medications or nutritional supplements. The study was cleared by the Research Ethics Board of Brock University (REB# 16-279), Canadian Sports Institute of Ontario (REB# 2017-01) and Health Canada’s—Natural and Non-prescription Health Products Directorate (NOA-229774), and has been registered with ClinicalTrials.gov (PRS; NCT04114045). The protocol and some of the measurements were previously described in Theocharidis et al. [18], which presented a parallel analysis of the effect of whey protein consumption on bone markers.

### 2.2. Design and Procedures

The study was carried out using a parallel, double-blind, placebo-controlled design. Participants were invited to the testing location for one information session and two testing sessions. In the information session, seated and standing height (cm) (SECA-217, CAN), as well as body mass (kg) and body fat percentage (InBody520, Biospace Co. Ltd., Madison, MI, USA) were recorded. Next, participants were stratified according to age, sex and body mass to one of the three experimental groups: whey protein (PRO), an isoenergetic nutritive control treatment of carbohydrate (CHO) or a non-nutritive control treatment of water (H_2_O), as described below. 

**Figure 1:**
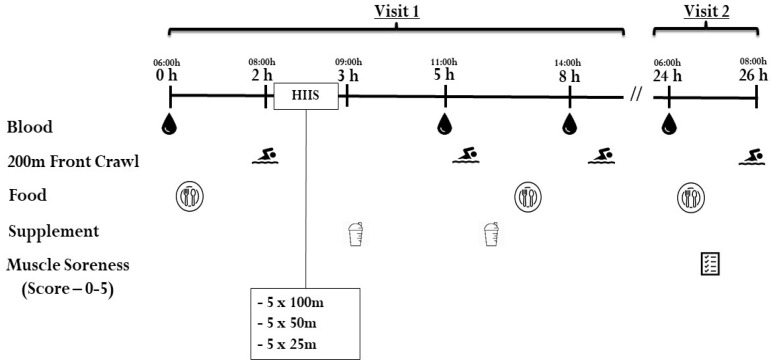
Study design and procedures. HIIS = high intensity interval swimming.

In addition, many of the female swimmers in each group were postmenarcheal (66% in PRO, 89% in CHO and 60% in H_2_O group). Since we could not schedule the testing in accordance with the phase of the individual (female) participants’ menstrual cycle, circulating estradiol concentrations were measured prior to the swimming trial to control for potential differences between the females in each group. All participants were instructed to refrain from exercise a minimum of 48 h prior to testing visit 1. An overview of the studies testing procedures (visits 1 and 2) can be found in Figure 1.

Testing visit 1 was scheduled one-week following the information session and was structured to mimic a competitive swim competition, with increased frequency of maximal effort swims at the beginning of the day, with fewer, more spread out swims as the day progressed to simulate quarterfinals, semi-finals and finals. Participants reported to the testing location at 05:30 h, where they then provided a fasted venous blood sample at 06:00 h (baseline, 0 h), followed by a standardized breakfast (described below). At ~1.5 h post-baseline (~1 h postprandial), participants began a warm-up of 1000 m in the testing pool (25 m). Subsequently, participants performed a maximal 200 m front-crawl swim, which served as their respective performance baseline. The 200 m front-crawl was chosen as it was an event most swimmers were familiar with and had best personal times to use as reference. Approximately 5 min after the performance test, participants performed a HIIS, which consisted of 5 × 100 m, 5 × 50 m and 5 × 25 m at >90% of each swimmer’s personal best time using a 1:1 work-to-rest ratio. Following the HIIS (~3 h post baseline), the first beverage was consumed. At ~5 h post baseline (~2 h post beverage consumption) participants provided another venous blood sample, then performed another maximal 200 m front-crawl swim and consumed the second beverage. Next, participants were provided with a low-protein standardized lunch (described below). At ~8 h post baseline (~3 h post beverage consumption), another venous blood sample was drawn followed by their final 200 m front-crawl swim test of the day. Prior to completing both the 5 and 8 h maximal 200 m front-crawl swims, participants performed a short warm-up of 500 m swim. During visit 2, 24 h following baseline measurements (06:00 h the following day), participants returned to the testing location where they provided a fasted venous blood sample, followed by the consumption of the same breakfast they received during visit 1. Following breakfast, participants were asked to rate their muscle soreness using an adapted Likert scale questionnaire [19]. At ~1 h postprandial participants began their 1000 m warm-up followed by the final maximal 200 m front-crawl swim. 

### 2.3. Nutrition

The standardized meals provided during visits 1 and 2 attempted to closely mimic typical race day nutrition patterns, avoiding heavy slow-digesting protein meals that may result in gastro-intestinal distress, followed by a larger dinner. Specifically, breakfast (visits 1 and 2) included one granola bar, one muffin, one fruit (banana or apple) and a juice box or water (carbohydrates = 80 g or 1.42–1.56 g/kg, protein = 5 g or 0.08–0.09 g/kg and fat = 20 g or 0.35–0.39 g/kg, ~400–500 kcal); lunch (visit 1) included one 12-inch veggie sandwich on a white bun, containing 1-slice of plain white cheese and any requested vegetables (carbohydrates = 88 g or 1.56–1.72 g/kg, protein = 8 g or ~0.14–0.15 g/kg and fat = 5 g or ~0.08–0.09 g/kg, ~400–500 kcal). The dinner between visits 1 and 2 was not standardized, alternatively participants were instructed to consume foods in similar quantities they normally would during a competitive swim meet to maintain ecological validity. Food consumption was monitored over the entire 24 h testing period (visits 1 and 2) using a self-reported diet record. To improve the precision of the diet record, participants and their parents were required to complete this record together to ensure accurate reporting (e.g., portion size) of food consumed. The food record was then analyzed using a diet analysis program (Food Processor, ESHA Inc., Salem, OR, USA), by the same examiner for consistency. Participants’ relative energy and macronutrient information is presented in Table 1.

### 2.4. Nutritional Supplement Protocol

Participants received two boluses of PRO, CHO or H_2_O with added non-energetic chocolate flavouring, served in black opaque shaker cups by an independent research assistant, to conceal the identity of the beverage’s contents to both the participants and the investigators (double blind). Participants consumed two servings of 0.3 g/kg of commercially available whey protein isolate (biPro, Eden Prairie, Eden Prairie, MN, USA), isoenergetic nutritive control beverage of carbohydrate (maltodextrin), or a non-nutritive beverage of flavoured water. Each beverage’s contents were dissolved in water (e.g., 1 g of whey protein isolate to 10 mL of water, as recommended by the manufacturer). The relative protein dose used in the present study (i.e., 0.3 g/kg/beverage) is based on previous findings by Moore and colleagues [20] who investigated the post-exercise response in whole body net protein balance in healthy children. Carbohydrates are generally more important for the maintenance of competition-level performance. However, the purpose of the present study was to investigate the effects of whey protein on recovery indices. Therefore, it is important to note that the carbohydrate beverage provided in the present study was meant as an isoenergetic placebo, and thus, the amount was below the recommended guidelines for repeated event sports (e.g., <0.8 g/kg/h) [21].

### 2.5. Blood Collection and Analysis

A total of 10 mL of blood was collected from an antecubital vein by a certified phlebotomist using a standard venipuncture technique at four time points: baseline, 5 h, 8 h and 24 h post baseline (Figure 1). All blood samples were centrifuged at 1405× *g* at 4 °C using a benchtop centrifuge for 10 min. Serum and plasma were then aliquoted into pre-labeled Eppendorf tubes and stored at −80 °C until analysis. 

Plasma concentrations of interleukins 6 and 10 (IL-6, IL-10), tumor necrosis factor alpha (TNF-α), creatine kinase (CK), as well as serum estradiol (in females) were measured in duplicate. Inflammatory cytokines were analyzed using Multiplex magnetic bead kits (Cat. #HSTCMAG-28SK, Milliplex EMD Millipore Corporation, Burlington, MA, USA). Creatine kinase was analyzed using a commercially available reagent kit (Cat. #C7522, Pointe Scientific Inc., Canton, MI, USA) fitted onto a 96-well plate and normalized with purified creatine kinase (Sigma, Oakville, ON, Canada, Cat. 10127566001). Estradiol was analyzed using a competitive ELISA kit (Human Estradiol E2 kit, Cat. ab108640, Abcam, Toronto, ON, Canada).

The average inter- and intra-assay coefficients of variation for IL-6, IL-10 and TNF-α were 9.2% and 5.8%, 5.7% and 5.4%, 6.4% and 6.4%, respectively. The average inter- and intra- assay coefficients of variation for CK were 4.5% and 3.6%, respectively. The average inter- and intra-assay coefficients of variation for estradiol were 5.2% and 8%, respectively.

*Hematocrit* was measured following all blood draws in triplicate by the same investigator using microhematocrit capillary tubes treated with heparin (VWR International, Radnor, PA, USA). Percent plasma volume changes (*%PV*) were calculated using the following formula by Van Beaumont [22].

$$\%\Delta PV=\left[\frac{100}{\left(100-Hematocrit_{Pre-exercise\;}\right)}\right]\times \left[\frac{100\left(Hematocrit_{Pre-exercise}-Hematocrit_{Post-exercise\;}\right)}{Hematocrit_{Post-exercise\;}}\right]$$

This formula was used to calculate %*PV* changes from pre-exercise across all time-points for each participant. The *%PV* changes were then used to adjust all post-exercise values for inflammatory mediators (IL-6, IL-10 and TNF-α) and CK.

### 2.6. Statistical Analysis

All statistical analyses were performed using IBM SPSS version 25 for windows (SPSS Inc., Chicago, IL, USA). The data are presented as means ±1 SE. There were no observed statistical differences across all variables between boys and girls. Therefore, data are expressed collectively. As previously mentioned, 60 participants were recruited for the study however, not all were included in the final analysis due to missing blood-draws (≥2 timepoints, *n* = 4) and outlying values (i.e., >3 SD, *n* = 2) leaving a total of 54 participants. Of the remaining 216 blood samples (54 participants × 4 sampling time points), two samples were missing due to unsuccessful venipuncture attempts at 5 h (in CHO) and 8 h (in H_2_O). As a result, these two participants were removed from the blood analysis. All data were assessed for normality by visual inspection of histograms, and by assessing the skewness and kurtosis ± 3 prior to parametric assessment. All variables met the assumptions of normality.

A one-way analysis of variance (ANOVA) was used to examine differences between groups in physical characteristics, estradiol concentrations (females only) and training history, as well as all other measures at baseline. Differences between groups with respect to muscle soreness was assessed using the Kruskal-Wallis test for non-parametric analysis. The changes in performance and in each of the biomarkers from baseline were examined using a two-way repeated measures ANOVA (RM-ANOVA), with one within-subject main-effect (time) and one between-subject main-effect (group). In the case of a significant main effect or interaction, pairwise comparisons were performed with Bonferroni adjustment for multiple comparisons. Cohen’s d effect sizes (ES) were calculated on absolute performance changes, where effects were considered small (0.2), medium (0.5), and large (>0.8) [23]. ESs are presented with the corresponding 95% confidence interval. The acceptable level of significance was set at *p* < 0.05 and *p* < 0.016 (0.05/3) for the Bonferroni adjustment.

## 3. Results

There were no significant differences between groups in age, physical characteristics, training history or among female’s estradiol concentration (Table 1). There were also no differences between groups in the relative macronutrient and energy consumption during the 24 h testing period (supplement not included) (Table 1).

**Figure 2:**
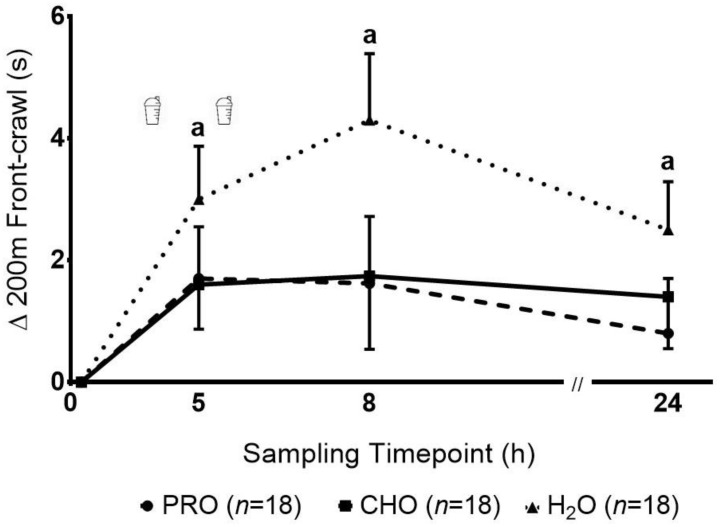
Changes in 200 m front crawl following a high intensity interval swimming protocol in adolescent swimmers. a—indicates a significant decrement (*p* < 0.016) in performance (e.g., longer performance times) compared to baseline in all groups.

There was a significant main effect of time (*p* = 0.001) observed in the 200 m swim performance, with no significant group effect (*p* = 0.15) or group-by-time interaction (*p* = 0.56), reflecting a reduction in performance (longer time to complete 200 m) following the HIIS in all groups (Figure 2). Pairwise comparisons revealed that 200 m swim performance was slower at 5 h, 8 h and 24 h post-HIIS compared to baseline. The ES and their corresponding 95% CI for the decrement in performance from baseline to 5, 8 and 24 h were lower, ranging from small to medium, in the PRO [0.7 (−0.1 to 3.5), 0.5 (−0.7 to 4) and 0.3 (−1 to 2.7), respectively] and CHO [0.7 (0.03 to 3.1), 0.5 (−0.9 to 4.4) and 0.6 (−0.3 to 3.2), respectively] groups, compared with the large ES observed in the H_2_O group [1.2 (1.2 to 4.8), 1.3 (2 to 6.6) and 1.1 (0.9 to 4.1), respectively].

**Figure 3:**
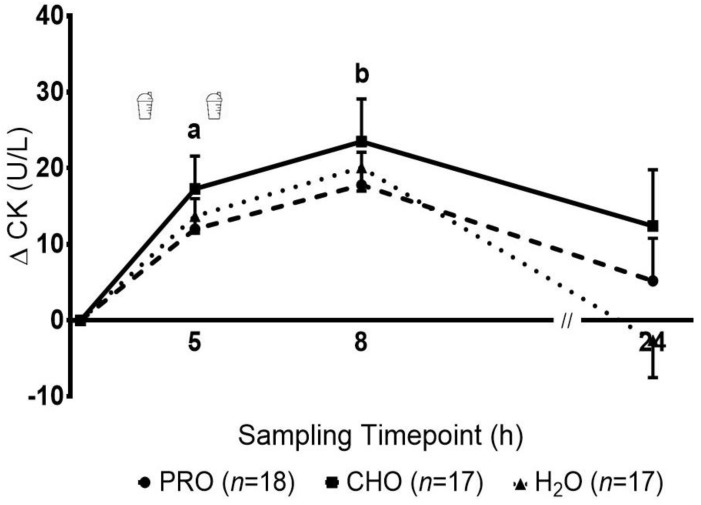
Changes in the plasma concentrations of creatine kinase (CK) following intense swimming in adolescent swimmers. a—indicates a significant increase (*p* < 0.016) at 5 h compared to baseline and 24 h in all groups. b—indicates a significant increase (*p* < 0.016) at 8 h compared with all other timepoints in all groups.

**Figure 4:**
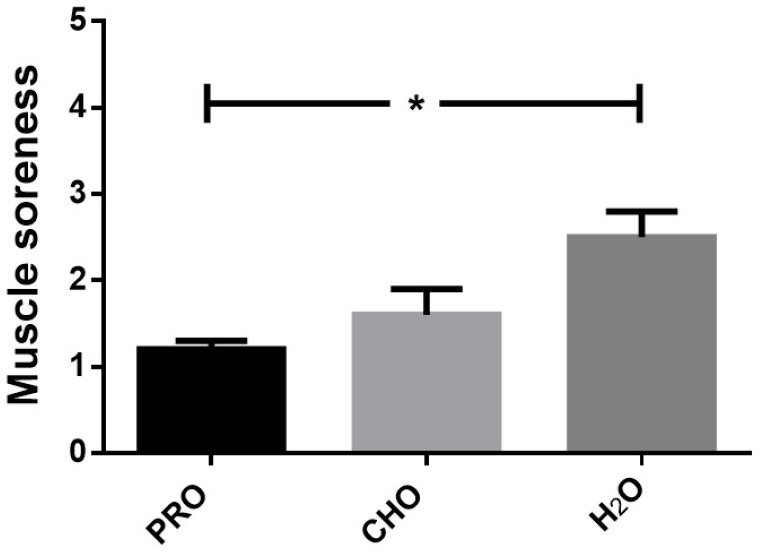
Perception of muscle soreness following intense swimming in adolescent swimmers. *—indicates a significant difference (*p* < 0.05) between PRO and H_2_O at 24 h.

The concentration of CK increased significantly over time (*p* = 0.001), with no significant group effect (*p* = 0.17) or group-by-time interaction (*p* = 0.25) (Figure 3). Pairwise comparisons revealed that CK at 5 h was significantly elevated compared to baseline and 24 h, and CK at 8 h was significantly elevated when compared to all other time points. Muscle soreness at 24 h was significantly different between groups (*p* = 0.04), with PRO significantly lower compared to H_2_O, but not CHO (Figure 4). No differences were observed between H_2_O and CHO. 

**Figure 5:**
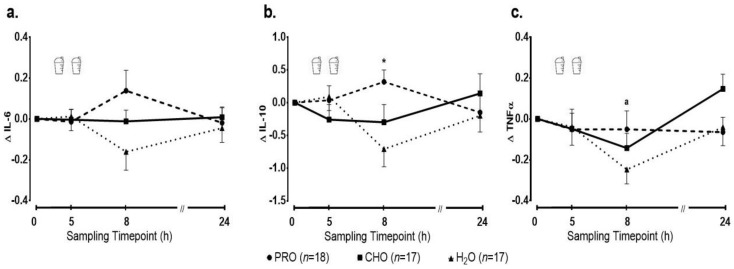
Changes in the plasma concentrations of (**a**) interleukin 6 (IL-6), (**b**) interleukin 10 (IL-10) and (**c**) tumor necrosis factor alpha (TNF-α) following intense swimming in adolescent swimmers. *—indicates a significant difference (*p* < 0.016) in IL-10 at 8 h post-exercise in PRO compared to H_2_O; a—indicates a significant decrease (*p* < 0.016) in TNF-α at 8 h compared to baseline and 24 h in all groups.

Baseline inflammatory cytokine levels are reported in Table 2. There was no significant main effect of time (*p* = 0.97) or group (*p* = 0.09), and no significant group-by-time interaction (*p* = 0.07) for the absolute change in IL-6 (Figure 5a). A significant group-by-time interaction (*p* = 0.005) was observed for IL-10, reflecting a significant increase in PRO at 8 h post-exercise, which was significantly different when compared with H_2_O (*p* = 0.02), but not when compared with CHO (*p* = 0.16) (Figure 5b). No significant main effect for time (*p* = 0.35) or group (*p* = 0.51) was observed. Lastly, absolute change in TNF-α from baseline following exercise indicated a significant main effect of time (*p* = 0.01). Pairwise comparisons indicated that TNF-α concentrations at 8 h were significantly lower compared to baseline and 24 h. No significant main effect for group (*p* = 0.45) or group-by-time interaction (*p* = 0.11) were observed (Figure 5c).

## 4. Discussion

The present study investigated the effect of whey protein consumption (2 × 0.3 g/kg) during the acute (0–8 h) and short-term (8–24 h) recovery periods in competitive adolescent swimmers. Overall, performance was not statistically different between groups. While the consumption of whey protein resulted in significant increases in anti-inflammatory IL-10 during the acute (0–8 h) recovery period, and a lower perception of muscle soreness at 24 h compared to the non-nutritive control group (H_2_O), no differences were observed when compared with the isoenergetic nutritive control treatment (CHO). 

Macronutrient consumption is an essential component in optimizing/hastening recovery and performance (e.g., refueling, rehydrating and repair/remodel) [7,16]. In the present study, we did not observe a statistically significant benefit to performance with the addition of either nutritive treatment protocols (e.g., whey protein or carbohydrate), compared to the non-nutritive consumption of water during the acute (0–8 h) nor the short-term (8–24 h) recovery periods. A possible explanation may relate to supplementation dosage. For example, participants in the present study consumed a similar or even greater relative protein dose (e.g., 0.3 g/kg) than that used in previous studies with adult athletes [4,24,25]. However, due to the increased demands of growth and development, accompanied by increased macronutrient needs associated with chronic training and competition, the protein dosages provided in the present study might not have been sufficient to significantly affect performance. Another explanation may relate to the athletes’ dietary intakes over the 24 h testing period. Specifically, protein (≥1.3 g/kg BM), carbohydrate (≥8 g/kg BM) and energy intakes (≥ ~50 Kcal/kg BM) over the 24 h testing period were either at or above the sports nutrition guidelines for youth athletes [26,27,28], which may have dampened any benefit of additional whey protein supplementation on performance. Evidence of this phenomenon has been observed in a meta-analysis where Schoenfeld et al. [29] observed that adults who consumed greater amounts of protein throughout the day, did not receive any added benefit (strength or hypertrophic response) from protein supplementation following intense exercise. However, most of the studies included within this meta-analysis were performed in untrained adults undergoing long-term resistance exercise programs focused on maximizing strength and muscle hypertrophy, which are not outcomes necessarily related to acute recovery. Future research in youth athletes is needed to determine the effects of higher doses of post-exercise protein supplementation, following high intensity exercise.

Subjective rating of muscle soreness and objective measures of skeletal muscle enzymes in blood (i.e., CK) were used in this study to indirectly reflect muscle damage and recovery. In the present study, small but significant elevations in plasma CK concentration were observed in all groups during both the acute and short-term recovery period, accompanied by increases in muscle soreness at 24 h. Together, these changes, along with the observed performance decrements suggest that HIIS resulted in some exercise-induced skeletal muscle damage. Whether whey protein was effective at attenuating muscle damage is difficult to ascertain. While the response in plasma CK concentration was similar in the three groups, perceived muscle soreness at 24 h post-HIIS was significantly lower in PRO compared with the non-nutritive control group H_2_O. Relative to adults, youth are characterized by lower peak concentration of CK [30]. Furthermore, the rise in CK concentration following intense swimming in both youth [31,32] as well as adults [15], appears to be lower (e.g., 60–300IU vs. 600–7000IU), peak sooner (e.g., 0–6 h vs. 24–48 h post-exercise) and returns to resting levels faster (e.g., 8 h vs. 48–72 h) than following eccentrically-based activities (e.g., resistance or plyometric exercise) [30,33,34]. Indeed, compared to eccentric activities, muscle damage (reflected in perceived soreness and CK response) observed in the present study appears to be considerably lower, suggesting that athletes would have less damage to recover from. Therefore, potential benefits of supplementation may have been too small to detect. Lastly, in the present study, we simulated a 1-day ecologically valid swim competition. However, it should be noted that among talented swimmers, these competitions can endure up to 8 days [1]. Multiple days of competition could potentially increase exercise-induced tissue damage, in which case protein supplementation may prove beneficial. 

Acute inflammation plays a critical role in the repair and regeneration of muscle [35]. Following exercise-induced muscle damage, the microenvironment is dominated by pro-inflammatory immune cells (e.g., neutrophils and M1 macrophages) to remove damaged tissue and debris [35]. These cells release pro-inflammatory mediators (e.g., TNF-α, IL-6) which facilitate the migration and proliferation of dormant satellite cells [36]. As M1 macrophages transition to M2, they release anti-inflammatory mediators (e.g., IL-10), which reduce inflammation and promote stem cell differentiation and fusion with injured muscle [35]. In the present study, we found no significant changes in IL-6 following HIIS, consistently across groups and irrespective of the higher baseline concentration in the H_2_O group. TNF-α concentrations at 8 h were significantly lower compared to baseline and 24 h in all groups, which may suggest an overall blunted inflammatory response in these adolescent swimmers. Importantly, we observed an increase in IL-10 at 8 h in PRO, which decreased in both CHO and H_2_O. The increase in IL-10 in the PRO group may reflect a protein-facilitated acceleration of the muscle regeneration process (e.g., anti-inflammatory mediators released earlier by immune cells). While previous studies with respect to protein consumption and its ability to modulate the inflammatory response are limited [9], our findings suggest that adolescent athletes respond similarly to post-exercise protein ingestion as previously described in adult athletes [10,11]. For example, Rowlands and colleagues [10] reported that in trained adults, a protein-leucine beverage consumed following intense cycling resulted in an attenuation of pro-inflammatory cytokines (IL-6, TNF-α) and the pathways that regulate them (nuclear factor kappa-β), which was not observed following consumption of an isoenergetic carbohydrate supplement. Similarly, using a cross-over design, Kerasioti et al. [11] observed in adults, that when a protein-carbohydrate cake was consumed following an intense cycling session, a ~50% reduction in pro-inflammatory cytokines (IL-6 and c-reactive protein) and a +110% higher response in IL-10 was observed compared to when participants consumed isoenergetic amounts of carbohydrate. Together, findings in the present and previous studies suggest a possible protein-facilitated inflammatory modulation. However, more research is needed to elucidate the meaningfulness of this mechanism with respect to performance recovery.

A limitation of the present study is that participants were not restricted in their dietary intake during dinner and were specifically instructed to consume the foods they normally would during a competition. As a result, potential differences in macronutrient consumption at dinner could affect both performance and biochemical results at 24 h, although there were no significant differences in macronutrient intake between groups at 24 h when supplementation was removed from both PRO and CHO. Additionally, the HIIS was designed in consultation with highly experienced swim coaches. However, it is possible that the prescribed HIIS was not sufficiently intense for all swimmers, especially the older, more experienced swimmers.

Despite the lack of statistical difference between groups with respect to performance, the present results may hold practical benefits to both coaches and swimmers. Specifically, during the acute recovery period (i.e., 0–8 h), where the time between events is more congested, the consumption of energy in the form of either whey protein or carbohydrate appears to offer some benefit. For example, the consumption of isoenergetic amounts of whey protein or carbohydrate showed better performance retainment (e.g., lowest time decrements) at 8 h (+1.62 s and +1.74 s respectively) compared with the non-nutritive control treatment of water (+4.26 s). In later stages of competition (e.g., ≥24 h) whey protein may offer additional benefits, reflected in lower perception of muscle soreness, which may translate into better performance retainment.

## 5. Conclusions

In conclusion, the consumption of two boluses of whey protein following a HIIS session did not result in significant performance recovery during both the acute (0–8 h) and short-term (8–24 h) recovery periods. While the consumption of whey protein resulted in significant increases in anti-inflammatory IL-10 during the acute recovery period (0–8 h), and a lower perception of muscle soreness at 24 h compared to the non-nutritive control group, no benefit was observed when compared with the isoenergetic nutritive control treatment of carbohydrate. Future research is required to further elucidate whey protein’s role in mediating inflammatory processes and how this may affect performance recovery in youth athletes.

## Figures and Tables

**Table 1:** Participants’ physical and training characteristics and 24 h energy and macronutrient consumption.

	Protein	Carbohydrate	Placebo
	**(*n* = 18)** **Boys = 9, Girls = 9**	**(*n* = 18)** **Boys = 9, Girls = 9**	**(*n* = 18)** **Boys = 8, Girls = 10**
Age (y)	13.4 ± 0.3	14.3 ± 0.4	14.0 ± 0.3
Years from age of PHV (y)			
Boys	0.1 ± 0.3	0.5 ± 0.4	0.3 ± 0.4
Girls	1.0 ± 0.2	1.9 ± 0.3	1.8 ± 0.5
Estradiol (pg/mL) Females only	10.7 ± 3.5	11.9 ± 2.2	8.6 ± 0.3
Height (cm)	160.4 ± 3.0	164.8 ± 2.3	165.2 ± 2.2
Body mass (kg)	51.0 ± 3.1	56.3 ± 2.6	55.1 ± 3.5
Body fat (%)	15.7 ± 1.4	16.1 ± 1.5	15.8 ± 1.8
Training History			
Years	4.6 ± 0.4	5.0 ± 0.4	4.7 ± 0.5
Sessions·wk^−1^	5.7 ± 0.3	6.3 ± 0.8	5.5 ± 0.3
24 h Energy Intake (kcal·kg^−1^)	55.1 ± 5.4	55.4 ± 4.2	49.3 ± 4.6
24 h Protein (g·kg^−1^)	1.9 ± 0.1 *	1.4 ± 0.2	1.3 ± 0.1
24 h Carbohydrate (g·kg^−1^)	8.5 ± 0.8	8.9 ± 0.6	8.1 ± 0.8

Values are mean ± standard error; PHV = Peak Height Velocity, 24 h energy and macronutrient consumption including supplements. * Indicates significant difference (*p* < 0.016) between protein and carbohydrate and protein and placebo. Total supplement contribution was included for protein (+0.6 g/kg) and carbohydrate (+0.6 g/kg).

**Table 2:** Baseline cytokine concentrations.

	Protein (*n* = 18)	Carbohydrate (*n* = 17)	Placebo (*n* = 17)
IL-6 (pg/mL)	0.9 ± 0.1	1.2 ± 0.1	1.5 ± 0.3 *
IL-10 (pg/mL)	4.2 ± 0.5	6.0 ± 0.5	5.6 ± 0.7
TNF-α (pg/mL)	1.9 ± 0.1	1.9 ± 0.1	2.1 ± 0.1

* Indicates significant difference (*p* < 0.05) between the placebo and protein groups.

## References

[1] Shaw G., Boyd K.T., Burke L.M., Koivisto A. (2014). Nutrition for swimming. Int. J. Sport Nutr. Exerc. Metab..

[2] Noakes T.D. (1987). Effect of Exercise on Serum Enzyme Activities in Humans. Sports Med..

[3] Burke L.M., Shaw G. (2014). Chapter 50 Swimming. The Encyclopedia of Sports Medicine.

[4] Brown M.A., Stevenson E.J., Howatson G. (2018). Whey protein hydrolysate supplementation accelerates recovery from exercise-induced muscle damage in females. Appl. Physiol. Nutr. Metab..

[5] Huang W.C., Chang Y.C., Chen Y.M., Hsu Y.J., Huang C.C., Kan N.W., Chen S.S. (2017). Whey protein improves marathon-induced injury and exercise performance in elite track runners. Int. J. Med. Sci..

[6] West D.W.D., Sawan S.A., Mazzulla M., Williamson E., Moore D.R. (2017). Whey protein supplementation enhances whole body protein metabolism and performance recovery after resistance exercise: A double-blind crossover study. Nutrients.

[7] Moore D.R., Camera D.M., Areta J.L., Hawley J.A. (2014). Beyond muscle hypertrophy: Why dietary protein is important. Appl. Physiol. Nutr. Metab..

[8] Nicastro H., Da Luz C.R., Chaves D.F.S., Bechara L.R.G., Voltarelli V.A., Rogero M.M.E., Lancha A.H. (2012). Does branched-chain amino acids supplementation modulate skeletal muscle remodeling through inflammation modulation? Possible mechanisms of action. J. Nutr. Metab..

[9] Torre-Villalvazo I., Alemán-Escondrillas G., Valle-Ríos R., Noriega L.G. (2019). Protein intake and amino acid supplementation regulate exercise recovery and performance through the modulation of mTOR, AMPK, FGF21, and immunity. Nutr. Res..

[10] Rowlands D.S., Nelson A.R., Raymond F., Metairon S., Mansourian R., Clarke J., Stellingwerff T., Phillips S.M. (2016). Protein-leucine ingestion activates a regenerative inflammo-myogenic transcriptome in skeletal muscle following intense endurance exercise. Physiol. Genom..

[11] Kerasioti E., Stagos D., Jamurtas A., Kiskini A., Koutedakis Y., Goutzourelas N., Pournaras S., Tsatsakis A.M., Kouretas D. (2013). Anti-inflammatory effects of a special carbohydrate-whey protein cake after exhaustive cycling in humans. Food Chem. Toxicol..

[12] Pennings B., Boirie Y., Senden J.M.G., Gijsen A.P., Kuipers H., Van Loon L.J.C. (2011). Whey protein stimulates postprandial muscle protein accretion more effectively than do casein and casein hydrolysate in older men. Am. J. Clin. Nutr..

[13] Pasiakos S.M., Lieberman H.R., McLellan T.M. (2014). Effects of protein supplements on muscle damage, soreness and recovery of muscle function and physical performance: A systematic review. Sports Med..

[14] Alghannam A.F., Gonzalez J.T., Betts J.A. (2018). Restoration of muscle glycogen and functional capacity: Role of post-exercise carbohydrate and protein co-ingestion. Nutrients.

[15] Cade J.R., Reese R.H., Privette R.M., Hommen N.M., Rogers J.L., Fregly M.J. (1991). Dietary intervention and training in swimmers. Eur. J. Appl. Physiol..

[16] Cintineo H.P., Arent M.A., Antonio J., Arent S.M. (2018). Effects of Protein Supplementation on Performance and Recovery in Resistance and Endurance Training. Front. Nutr..

[17] Aerenhouts D., Deriemaeker P., Hebbelinck M., Clarys P. (2011). Energy and macronutrient intake in adolescent sprint athletes: A follow-up study. J. Sports Sci..

[18] Theocharidis A., McKinlay B.J., Vlachopoulos D., Josse A.R., Falk B., Klentrou P. (2020). Effects of post exercise protein supplementation on markers of bone turnover in adolescent swimmers. J. Int. Soc. Sports Nutr..

[19] Vickers A.J. (2001). Time course of muscle soreness following different types of exercise. BMC Musculoskelet. Disord..

[20] Moore D.R., Volterman K.A., Obeid J., Offord E.A., Timmons B.W. (2014). Postexercise protein ingestion increases whole body net protein balance in healthy children. J. Appl. Physiol..

[21] Van Loon L.J.C., Saris W.H., Kruijshoop M., Wagenmakers A.J.M. (2000). Maximizing postexercise muscle glycogen synthesis: Carbohydrate supplementation and the application of amino acid or protein. Am. J. Clin. Nutr..

[22] Van Beaumont W. (1972). Evaluation of hemoconcentration from hematocrit measurements. J. Appl. Physiol..

[23] Cohen J. (1977). Statistical Power Analysis for the Behavioral Sciences.

[24] Buckley J.D., Thomson R.L., Coates A.M., Howe P.R.C., DeNichilo M.O., Rowney M.K. (2010). Supplementation with a whey protein hydrolysate enhances recovery of muscle force-generating capacity following eccentric exercise. J. Sci. Med. Sport.

[25] Daly R.M., O’Connell S.L., Mundell N.L., Grimes C.A., Dunstan D.W., Nowson C.A. (2014). Protein-enriched diet, with the use of lean red meat, combined with progressive resistance training enhances lean tissue mass and muscle strength and reduces circulating IL-6 concentrations in elderly women: A cluster randomized controlled trial. Am. J. Clin. Nutr..

[26] Burke L., Deakin V. (2015). Clinical Sports Nutrition.

[27] Meyer F., O’Connor H., Shirreffs S.M. (2007). Nutrition for the young athlete. J. Sports Sci..

[28] Purcell L.K. (2013). CPS Practice Point: Sport nutrition for young athletes. Paediatr. Child Health.

[29] Schoenfeld B.J., Aragon A., Krieger J.W. (2013). The effect of protein timing on muscle strength and hypertrophy: A meta-analysis. J. Int. Soc. Sports Nutr..

[30] Chen T.C., Chen H.L., Liu Y.C., Nosaka K. (2014). Eccentric exercise-induced muscle damage of pre-adolescent and adolescent boys in comparison to young men. Eur. J. Appl. Physiol..

[31] Dabidi Roshan V., Babaei H., Hosseinzadeh M., Arendt-Nielsen L. (2013). The effect of creatine supplementation on muscle fatigue and physiological indices following intermittent swimming bouts. J. Sports Med. Phys. Fit..

[32] Fu F.H., You C.Y., Kong Z.W. (2002). Acute changes in selected serum enzyme and metabolite concentrations in 12-to 14-yr.-old athletes after an all-out 100-m swimming sprint. Percept. Mot. Ski..

[33] Peake J.M., Neubauer O., Gatta P.A.D., Nosaka K. (2017). Muscle damage and inflammation during recovery from exercise. J. Appl. Physiol..

[34] Clarkson P.M., Hubal M.J. (2002). Exercise-Induced Muscle Damage in Humans. Am. J. Phys. Med. Rehabil..

[35] Yang W., Hu P. (2018). Skeletal muscle regeneration is modulated by inflammation. J. Orthop. Transl..

[36] Peterson J.M., Bakkar N., Guttridge D.C. (2011). NF-κB signaling in skeletal muscle health and disease. Curr. Top. Dev. Biol..

