# Correction: McKinlay et al. Effects of Post-Exercise Whey Protein Consumption on Recovery Indices in Adolescent Swimmers. *Int. J. Environ. Res. Public Health* 2020, *17*, 7761

**DOI:** 10.3390/ijerph192316311

**Published:** 2022-12-06

**Authors:** Brandon J. McKinlay, Alexandros Theocharidis, Tony Adebero, Nigel Kurgan, Val A. Fajardo, Brian D. Roy, Andrea R. Josse, Heather M. Logan-Sprenger, Bareket Falk, Panagiota Klentrou

**Affiliations:** 1 Department of Kinesiology, Faculty of Applied Health Sciences, Brock University, St. Catharines, ON L2S 3A1, Canada; 2 Centre for Bone and Muscle Health, Faculty of Applied Health Sciences, Brock University, St. Catharines, ON L2S 3A1, Canada; 3 School of Kinesiology and Health Science, Faculty of Health, York University, Toronto, ON M3J 1P3, Canada; 4 Canadian Sport Institute Ontario, 857 Morningside Avenue, Toronto, ON M1C 0C7, Canada; 5 Faculty of Health Sciences, Ontario Tech University, Oshawa, ON L1G 0C5, Canada

The authors of “Effects of Post-Exercise Whey Protein Consumption on Recovery Indices in Adolescent Swimmers” report an error in Table 1 of their article [1]. The somatic maturity estimation for the female swimmers was mistakenly based on the male equations. Thus, the somatic maturity of the female swimmers was re-calculated using the female-specific equation by Mirwald et al. [2]. The corrected table appears below. The corrected values do not change the results and scientific conclusions of the article.

## Figures and Tables

**Table 1:** Participants’ physical and training characteristics and 24 h energy and macronutrient consumption.

	Protein	Carbohydrate	Placebo
	(*n* = 18) Boys = 9, Girls = 9	(*n* = 18) Boys = 9, Girls = 9	(*n* = 18) Boys = 8, Girls = 10
Age (y)	13.4 ± 0.3	14.3 ± 0.4	14.0 ± 0.3
Years from age of PHV (y)			
Boys	0.1 ± 0.3	0.5 ± 0.4	0.3 ± 0.4
Girls	1.0 ± 0.2	1.9 ± 0.3	1.8 ± 0.5
Estradiol (pg/mL) Females only	10.7 ± 3.5	11.9 ± 2.2	8.6 ± 0.3
Height (cm)	160.4 ± 3.0	164.8 ± 2.3	165.2 ± 2.2
Body mass (kg)	51.0 ± 3.1	56.3 ± 2.6	55.1 ± 3.5
Body fat (%)	15.7 ± 1.4	16.1 ± 1.5	15.8 ± 1.8
Training History			
Years	4.6 ± 0.4	5.0 ± 0.4	4.7 ± 0.5
Sessions·wk^−1^	5.7 ± 0.3	6.3 ± 0.8	5.5 ± 0.3
24 h Energy Intake (kcal·kg^−1^)	55.1 ± 5.4	55.4 ± 4.2	49.3 ± 4.6
24 h Protein (g·kg^−1^)	1.9 ± 0.1 *	1.4 ± 0.2	1.3 ± 0.1
24 h Carbohydrate (g·kg^−1^)	8.5 ± 0.8	8.9 ± 0.6	8.1 ± 0.8

Values are mean ± standard error; PHV = Peak Height Velocity, 24 h energy and macronutrient consumption including supplements. * Indicates significant difference (*p* < 0.016) between protein and carbohydrate and protein and placebo. Total supplement contribution was included for protein (+0.6 g/kg) and carbohydrate (+0.6 g/kg).
